# Synthesis and robocasting of YAG xerogel: one-step conversion of ceramics

**DOI:** 10.1038/s41598-022-12204-6

**Published:** 2022-05-19

**Authors:** Nancy Flores-Martinez, Lila Ouamara, Fabien Remondiere, Jenny Jouin, Giuseppe Fiore, Stephane Oriol, Sylvie Rossignol

**Affiliations:** 1 Centre National d’Etudes Spatiales, Direction des Lanceurs, 52 rue Jacques Hillairet, 75615 Paris Cedex, France; 2 Institut de Recherche sur les Céramiques, UMR 7315, 12 rue Atlantis, 87068 Limoges Cedex, France

**Keywords:** Engineering, Materials science, Chemistry

## Abstract

An optimized sol–gel protocol was carried out to produce an yttrium aluminum garnet (YAG) xerogel from aluminum alkoxide and an yttrium salt on a semi-pilot scale. This xerogel was successfully used without prior pyrolysis as a solid load with the aid of additives in the preparation of pastes. Thermal treatment of the green bodies, obtained by robocasting of the paste, led to cohesive single-phase YAG ceramics. Manufacturing ceramic pieces by additive methods will allow shaping complex forms, while the single step conversion/consolidation would simplify the technological process, reducing global energy costs. Since YAG possesses high strength and good creep behavior at high temperatures, these refractory pieces could replace the metal alloys used in turbine blades for deep space exploration. Structural, thermal and chemical characterizations were performed on xerogel powders, pastes, and YAG ceramics.

## Introduction

The French Space Agency (CNES) has carried out research and development into oxide ceramics with the aim of improving the design of crucial subsystems for space propulsion. The maximum allowable turbine temperature, imposed by the resistance of metallic alloys, represents a performance limitation for liquid propulsion rocket engine cycles. The introduction of oxide ceramics for stator/rotor turbine parts could be a promising solution to increase the cycle temperature and achieve performance gains accordingly. From a lifetime standpoint, creep-resistant ceramics would be the key technology for the development of onboard power production systems for deep-space exploration^1^. Yttrium aluminum garnet (YAG, Y_3_Al_5_O_12_) was chosen for this purpose. Besides being known as a laser gain host material^2–4^ for solid-state lasers^5^, it can also be utilized for its mechanical characteristics. Indeed, it presents interesting mechanical properties at high temperature^6^, due to its high strength, good creep behavior at high temperatures (> 1000 °C), good physical and chemical stability, low thermal conductivity, and good water vapor corrosion resistance^7^. It is also used in oxidizing environments for thermal barrier coatings^8^ or in applications requiring long-term retention^9^ as well.

Among all the reported protocols for YAG preparation including solid-state^10,11^, sol–gel-based synthesis has proven to be a good method to prepare single-phased YAG, as the homogeneous mixing of precursors in the sol–gel method guarantees the chemical uniformity of the product and a lower processing temperature^12^. For example, following this process, Gowda^13^ prepared gels of yttria and aluminum tri-*sec*-butoxide acetate, which crystallized into YAG when thermally treated between 800 and 1400 °C. Furthermore, Manalert and Rahaman^14^ obtained amorphous YAG from a mixture of aluminum tri-*sec*-butoxide and yttrium acetate hydrate using the sol–gel method and supercritical drying with extraction of CO_2_. Finally, Singlard et al*.*^15^ developed a sol–gel synthesis of single-phased YAG from aluminum tri-*sec*-butoxide and anhydrous yttrium chloride and subsequent heat treatment.

In any case, these powders must be manufactured and shaped while maintaining their properties as ceramics. Currently, due to its low cost and ease of use, extrusion is one of the most widely used technologies for the direct shaping of ceramics^16,17^. In the case of YAG manufacturing, just a few examples can be found in the literature, namely the 3D printing using a mixed powder aqueous slurry^18^ and the 3D direct ink writing of YAG nanoparticles^19^. However, most of these innovations belong to the optics field, where YAG is doped with rare-earth metal elements and the desired properties are related to refractive index^17^, photoluminescence^20^, etc. and nothing is dealing with the extrusion of xerogel.

From a technological point of view, the 3D printing process requires a large quantity of solid load. Nevertheless, as often mentioned in the literature, chemical routes for YAG powders tend to be limited to laboratory-scale quantities, and it could be a challenge to yield larger amounts. Scaling up YAG powder production is far from straightforward, as enlarging can lead to the formation of impurities, influence the reproducibility of the process, or alter the microstructure of the products. Moreover, the direct use of xerogel as a solid load in the paste can offer an alternative way to simplify the heat treatment profile. Indeed, it is possible to take advantage of the debinding step to promote the conversion of the xerogel into crystalline YAG, avoiding the usual prepyrolysis of the xerogel.

The aim of this study was to improve and scale up the process for preparing a YAG xerogel. Then, the printability of the xerogel-based paste was studied to shape consolidated YAG pieces in one-step process. Thermal, structural, and microstructural characterizations were performed on the samples.

## Materials preparation

### Scaling up the sol–gel synthesis of YAG xerogel and YAG powder

**Figure 1:**
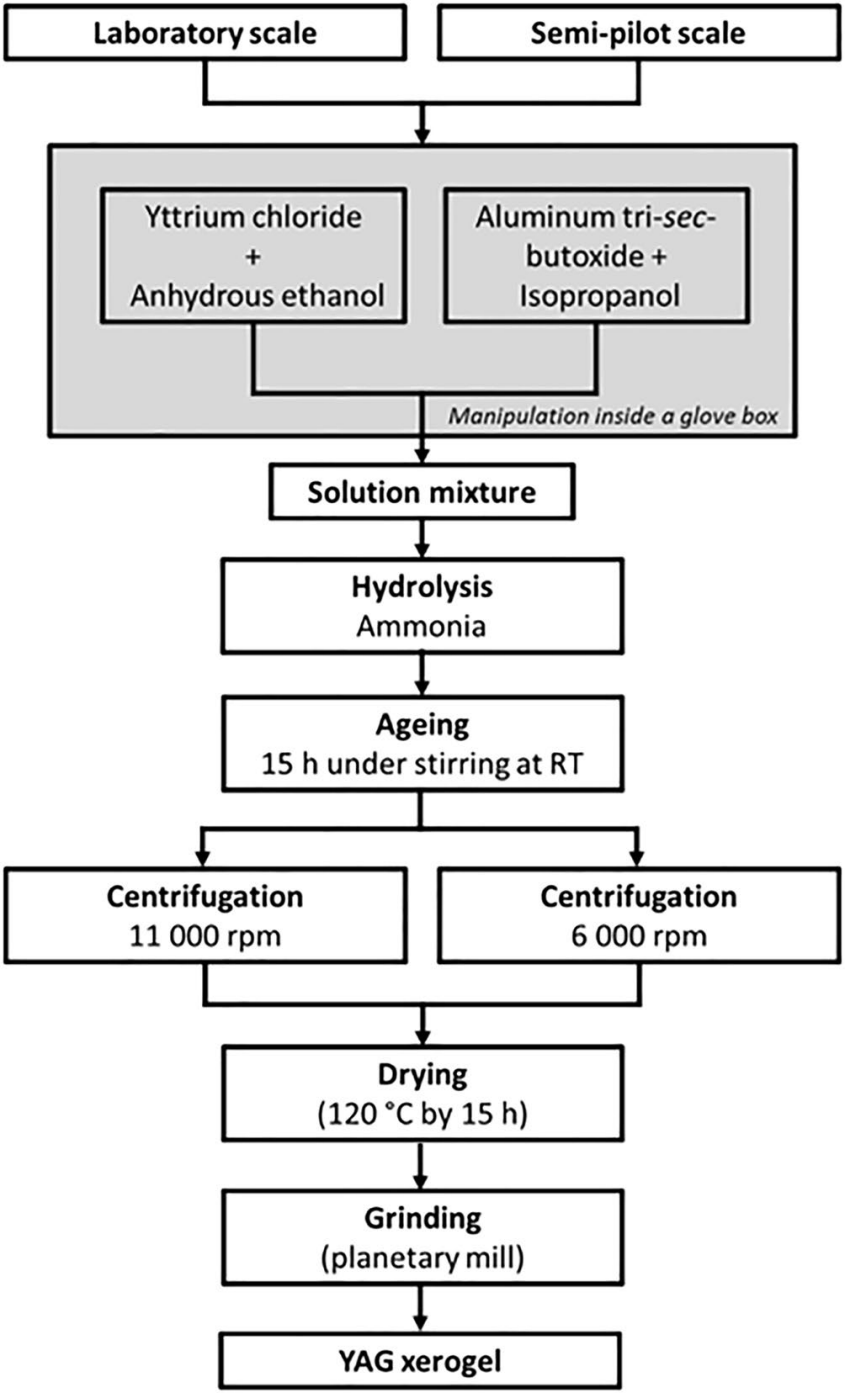
Preparation protocol for L and SP xerogels.

The metal precursors used for the sol–gel synthesis were anhydrous yttrium chloride (99.99%, Sigma–Aldrich) and aluminum tri-*sec*-butoxide (97%, Sigma–Aldrich), while the solvents used were anhydrous ethanol (94–96%, Alfa Aesar) and isopropanol (99.9% Fisher Scientific). For hydrolysis, ammonia (28%, Alfa Aesar) was employed. We produced YAG xerogel following the protocol described by Singlard et al.^15^ but decreasing the maturation temperature from 60 °C to room temperature. This protocol was denoted “laboratory scale synthesis” and noted as “L”. For enlarging the production of xerogel, keeping the same characteristics of xerogel produced by L, a second protocol called “semi-pilot scale synthesis” and named “SP” was carried out. In this protocol 0.27 mol of yttrium chloride was dissolved in 330 mL of anhydrous ethanol. On the other hand, 0.25 mol of aluminum tri-sec-butoxide was dissolved in 330 mL of isopropanol. Both solutions were mixed, in a 2L-reactor, inside a glove box, especially to preserve the anhydrous character of the yttrium chloride powder. Then, hydrolysis was completed consuming 83 mL ammonia as catalyzer. The solution was aged during 15 h at room temperature to mature the sol and centrifuged at 6 000 rpm. A detailed protocol for L and SP is shown in Fig. 1. For both syntheses, three washes in deionized water were necessary. The centrifuged xerogel was dried at 120 °C/15 h under 115 mbar of pressure. To verify that the YAG phase is formed from L and SP xerogels, a subsequent calcination was performed. L and SP were heated in a first step at 300 °C for 2 h with a heating rate of 2 °C/min followed by a second step at 1000 °C for 1 h with a heating rate of 5 °C/min and finally natural cooling. After calcination, the sample corresponding to L was called L1000, and that for SP was named SP1000.

### Preparation and extrusion of YAG xerogel pastes

The YAG xerogel paste designed for extrusion is composed of a mixture of SP xerogel as the solid load and poly vinyl alcohol, PVA (Rhodoviol 25/140, VWR chemicals, Leuven, Belgium), in aqueous solution (97 g/L) as the unique additive. The paste was prepared as follows: a specific volume of polyvinyl alcohol solution was vigorously mixed with 68.75 wt% SP xerogel, resulting in the formation of a slurry that was stirred until a homogeneous paste was obtained.

The paste was then extruded to form cords’ structures. Before any heat treatment, the extruded pieces were exposed to 50% relative humidity (RH) for at least 15 h at room temperature. Then, the pieces were debinded at 600 °C for 2 h with a heating rate of 2 °C/min to eliminate the aqueous polyvinyl alcohol additive and organic remnants from the sol–gel synthesis.

**Figure 2:**
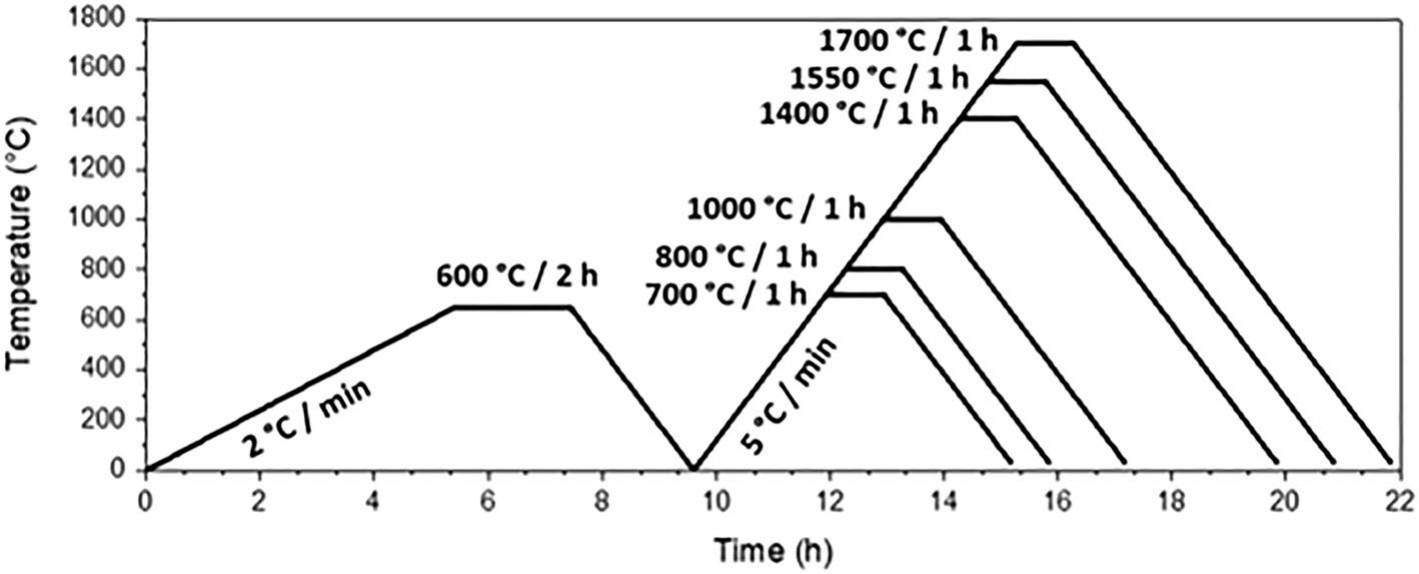
Thermal treatments for the extruded pastes prepared with YAG xerogel SP.

Finally, these pieces were treated at 700 °C, 800 °C, 1000 °C, 1400 °C, 1550 °C and 1700 °C. All these thermal treatments were performed with a dwelling time of 1 h following a heating rate of 5 °C/min under static conditions, as shown in Fig. 2.

## Results and discussions

### Scaling-up the YAG xerogel sol–gel synthesis

**Figure 3:**
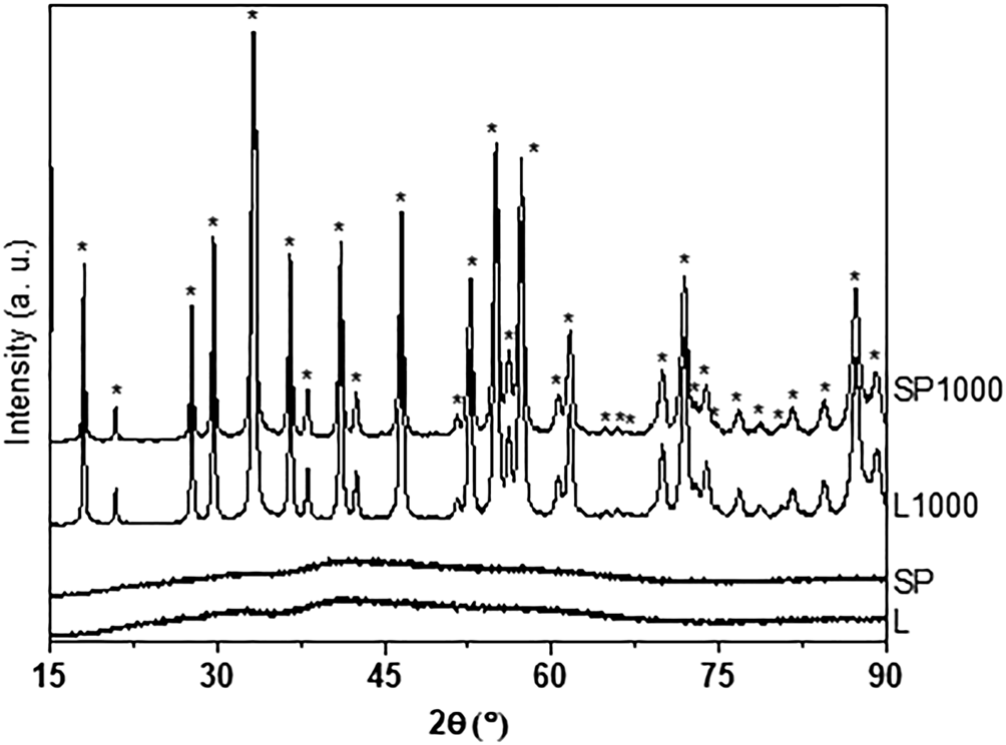
Dried (L, SP) and calcined (L1000, SP1000) YAG xerogel XRD patterns. (*) Peaks corresponding to the ICSD reference card for Y_3_Al_5_O_12_, PDF 04-007-2667.

The diagrams resulting from X-ray diffraction analysis are shown in Fig. 3 for the L and SP xerogels, as well as calcined L1000 and SP1000, thus allowing to compare the powders obtained following the two synthesis methods. As expected, L and SP xerogels are amorphous. When calcined at 1000 °C (L1000 and SP1000), the organic residues were eliminated, leading to a polycrystalline ceramic powder. According to the PDF file 04-007-2667, both XRD patterns for the calcined xerogels match with a pure YAG structure, without any notable extra phase. Even if the synthesis protocol was slightly modified for L, compared to that of Singlard’s and collaborators^15^, we observe the same features for the xerogel and calcined xerogel.

**Figure 4:**
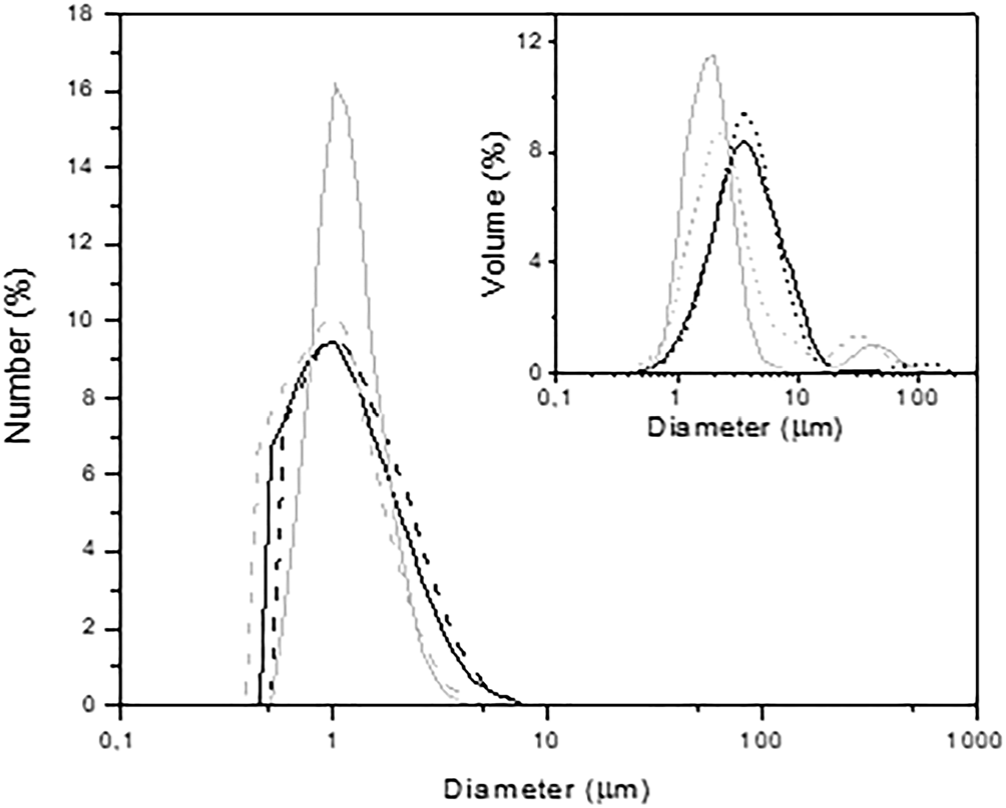
Number distribution of particles for dried (black) and calcined (gray) xerogel powders for the L (---) and SP (—) protocols. Inset correspond to the volume distribution of the particles.

Figure 4 displays the particle size distribution in number for L, SP, L1000 and SP1000 samples. In all cases, these distributions are very similar; there is a single population between 2 and 3 µm of diameter. D_50_ and D_90_ values can be found in the Table 1. Regarding the volume distribution, in the inset, the presence of few, larger agglomerates (greater than 30 µm) is evidenced. The low probability of presence of these agglomerates was verified during the extrusion of the SP-based cords since the nozzle was not clogged and a clear printing of the YAG xerogel cords was performed.

**Table 1:** Measured density, D_50_ and D_90_ corresponding to the number distribution for the dried and calcined xerogels.

YAG xerogel	Sample name	Density ± 0.05 (g/cm^3^)	D_50_/D_90_ ± 0.1 (µm)
Dried (120 °C)	L	2.23	3.4/7.4
	SP	2.20	3.4/8.1
Calcined (1000 °C)	L1000	4.47	2.3/3.4
	SP1000	4.46	1.8/3.9

Finally, the density of the powders is another important parameter to consider when scaling up the sol–gel synthesis. Table 1 displays the density values for the xerogels and calcined samples. The densities of the xerogels were approximately 2.20 g/cm^3^, whether prepared with the laboratory or the enlarged scale synthesis protocols. This relatively low value is due to the high amount of organic phase in the samples, which appears to be similar for both cases. After calcination at 1000 °C, the density of the samples reached 4.47 g/cm^3^ and 4.46 g/cm^3^ for L1000 and SP1000, respectively. This increment is due to the thermal conversion of the xerogel into an inorganic network. Once the organic residues were eliminated, the amorphous phase was allowed to crystallize into the YAG structure, which does not mean that the arrangement between the grains was optimized. Nevertheless, considering the measurement errors and the YAG theoretical density value of 4.55 g/cm^3^, it can be noted that the relative differences between the theoretical and experimental densities are 1.8% for L1000 and 1.9% for SP1000, meaning that the samples are close to pure YAG.

In conclusion, both xerogels led to the formation of pure single-phase YAG samples upon calcination at 1000 °C, regardless of the scale (L or SP) of the syntheses. Moreover, the particle size distribution and density of xerogels and powders possess similar characteristics as well, which confirms relatively good similarities for the products after scaling up the laboratory scale procedure and allows the use of a semi-pilot scale synthesis for the subsequent preparation of pastes and ceramics.

### Preparation and thermal behavior of the xerogel paste

**Figure 5:**
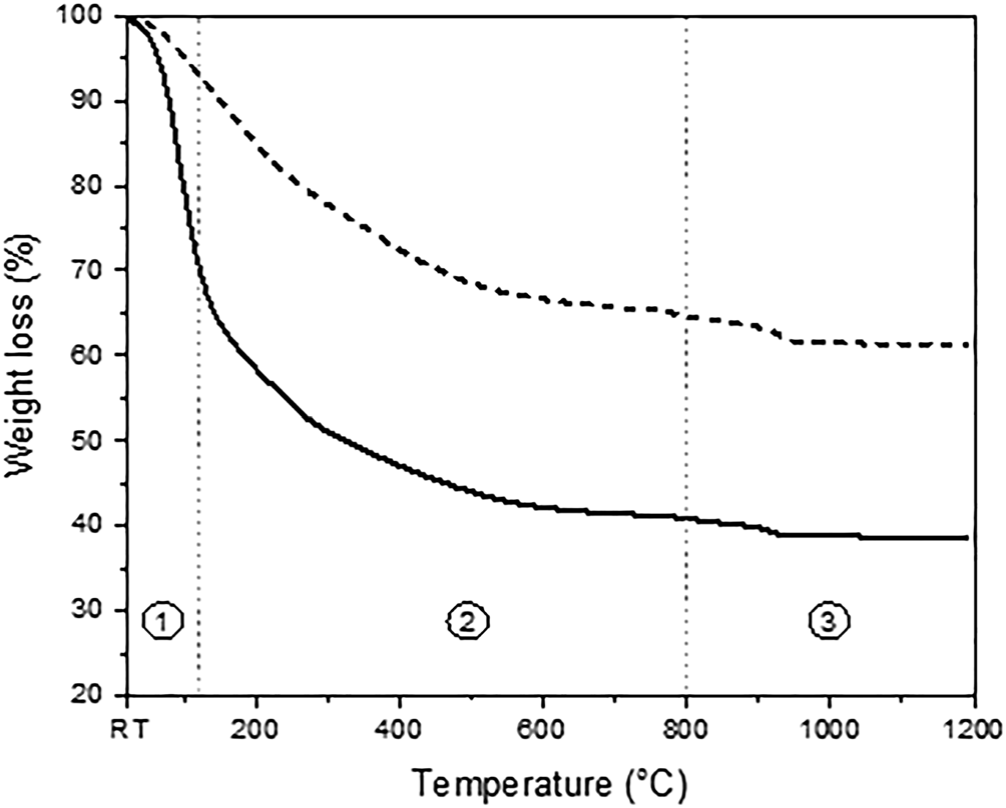
Thermograms for SP powder (---) and SP based paste (—). Three zones can be distinguished corresponding to: ① evaporation, ② decomposition and ③ decarbonization processes.

In all the following experiments, the SP xerogel was used as the solid load in the paste formulation. The powder and paste thermal behaviors were thus studied through thermal analyses, as shown in Fig. 5 (full thermograms are provided in the additional information section), which displays the weight loss for the xerogel and for the paste prepared with SP and aqueous polyvinyl alcohol solution. The powder, denoted for a dotted line, exhibits a global weight loss of 38.6%, which can be divided into three zones. The first zone, from 20 to 120 °C, presents a loss of 6.8% associated with the evaporation of organic solvents and water. The second zone, between 120 and 800 °C, exhibits a weight loss of 28.5%. It is well known that between 200 and 500 °C, decomposition and/or combustion of organic residues occurs. The final zone, from 800 to 1200 °C, corresponds to a very small weight loss of 3.3%. This can be connected to residual decarbonization and crystallization of the amorphous network into the YAG structure, as already shown in Fig. 3. The different weight losses are in good agreement with the results reported by Singlard et al*.*^15^ in terms of the number of defined zones and the nature of related thermal events. On the other hand, the thermogram for the paste, denoted by a continuous line, shows the same global features as observed in the powder thermogram. However, the total weight loss is largely increased to 61.3%, since the sample contains aqueous polyvinyl alcohol in addition to the organic residues issued from the sol–gel synthesis. The three weight losses for the first, second and third zones are 29.4%, 29.7% and 2.2%, respectively. Thus, the presence of aqueous polyvinyl alcohol does modify essentially the evaporation and decomposition zones and barely affects the last decarbonization/crystallization event.

### Extrusion and thermal transformation of the xerogel paste

**Figure 6:**
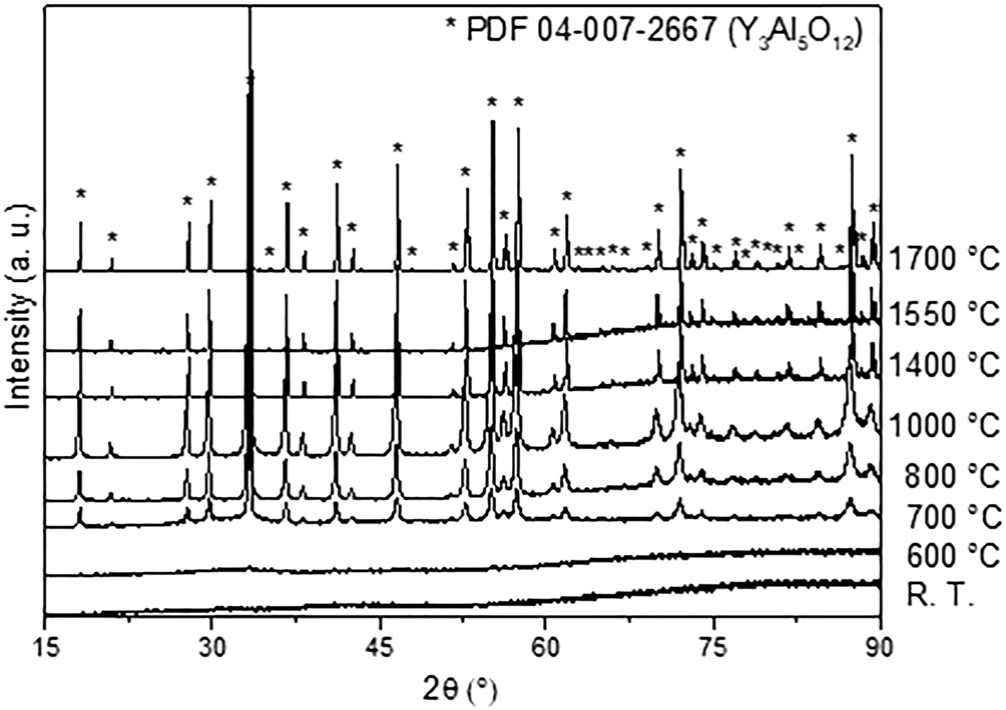
XRD patterns for the cords obtained from the paste prepared from YAG xerogel SP after calcination in air at different temperatures.

XRD patterns for the extruded cords calcined at different temperatures are shown in Fig. 6. At room temperature, no peak is clearly observed, as the samples exhibit an amorphous structure characteristic of xerogels. At 600 °C, the amorphous phase remains predominant. However, once the temperature reaches 700 °C, crystallization occurs. The same XRD reflections become more defined and intense at 800–1000 °C. Using the reference card PDF n°04-007-2667, it was found that all the peaks can be indexed with respect to the garnet structure. From 1400 up to 1700 °C, the same reflections are visible, although they appear to be much sharper. However, between 1550 and 1700 °C, the presence of impurities is barely distinguished in the X-ray patterns. The identification of this minor impurity is not possible, as it is mostly present as peak shoulders and very low intensity signatures. One has to keep in mind that despite the purity of the aluminum precursor, yttrium aluminum monoclinic, YAM, and yttrium aluminum perovskite, YAP, intermediate phases were reported to form during YAG synthesis, and to co-exist after prolonged heating in the range between 1000 and 1800 °C^21,22^.

Moreover, the broadening of the main peak (4 2 0) in the diagrams was measured from 700 to 1700 °C to further investigate the ordering of the YAG structure in the particles. These results are gathered in Table 2, which shows that the broadening is quite stable at 0.4° up to 1000 °C. Then, starting at 1400 up to 1700 °C, a sharp decrease in the (4 2 0) peak down to 0.1° is observed suggesting a better organization of the YAG and the presence of a lower amount of microstructural defects in these samples. In summary, the formation of garnet from an amorphous inorganic network obtained after complete combustion of the organic residue and polyvinyl alcohol content starting at 700 °C is achieved at 1000 °C. Then, between 1000 and 1400 °C, an increase in the size of the coherent domains is observed, which shows an activation of the material diffusion and a decrease in the density of defects. The broadening between 1400 and 1700 °C is stable at around 0.1° which does not give more information about the organization of coherent domains.

**Table 2:** Broadening of the (4 2 0) main peak, as extracted from the XRD patterns, as a function of the calcination temperature.

Temperature (°C)	Broadening (°)
700	0.37(1)
800	0.41(9)
1000	0.37(1)
1400	0.11(1)
1550	0.09(1)
1700	0.08(2)

**Figure 7:**
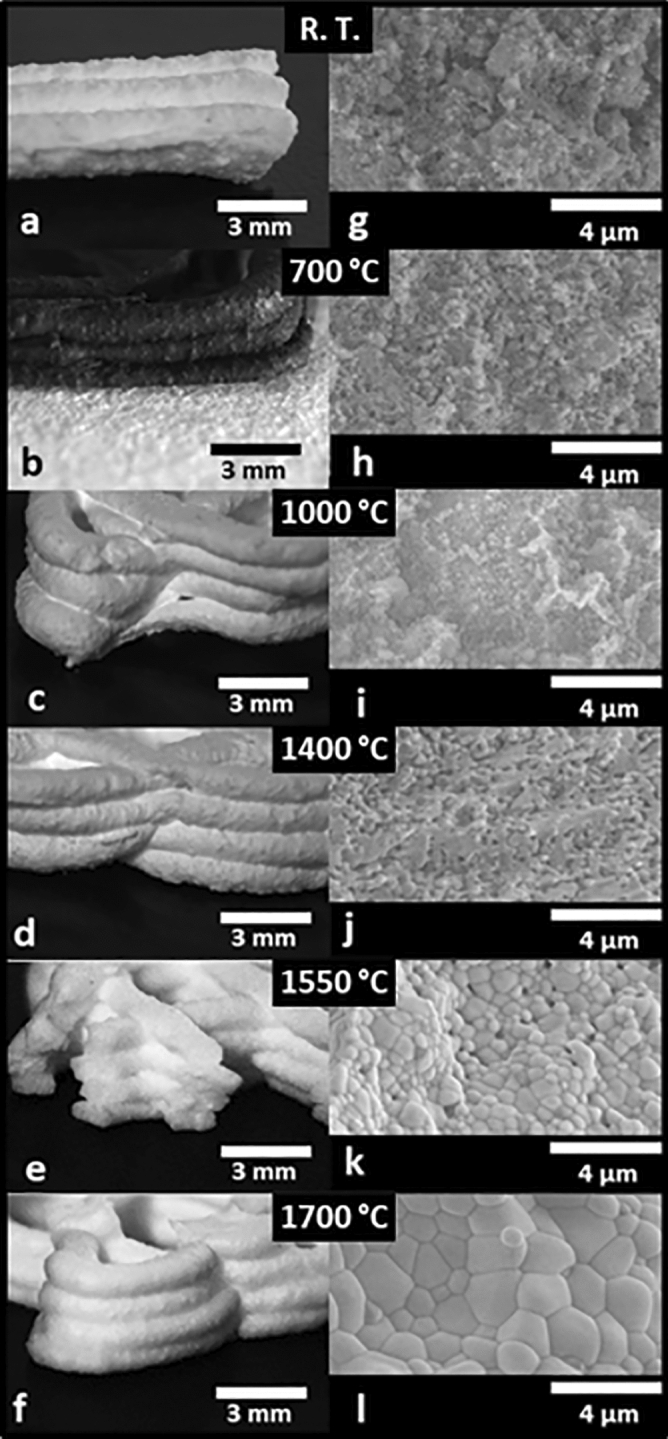
High-resolution images (**a**-**f**) and SEM micrographs (**g**-**l**) of fractured cords at different temperatures.

Furthermore, high-resolution images of the layer-by-layer manufacturing cords calcined at different temperatures were taken, Fig. 7(a-f). These captures showed the stacking of the printed paste. Definition, shape, and consistence of the as-printed cords (a) are retained even after debinding (b), calcination (c-d) and consolidation (e-f) steps. Note that at 700 °C the presence of carbon residues is visible in the dark gray color of the sample. To better observe the evolution of their microstructure, SEM micrographs of fractured cords were analyzed, Fig. 7(g-l). From room temperature (g) to 700 °C (h), the microstructure is typical of a xerogel with poorly arranged small grains. At 1000 °C (i), the packing of the grains improves, however they remain quite small. In the temperature range from 1400 to 1700 °C (j-l), the better crystallization of the grains, suggested from the lower broadening of the XRD peaks, is visible as their size increases up to 2 µm at 1700 °C.

Underlining that the fresh paste was formulated from an aqueous poly vinyl alcohol solution and xerogel, it should be noted that the global cohesion between the printed cords was attained in the green pieces and was retained after thermal treatments. The sintering of the material was also found to be effective between 1550 and 1700 °C since the coalescence was thermally activated without abnormal grain growth.

**Figure 8:**
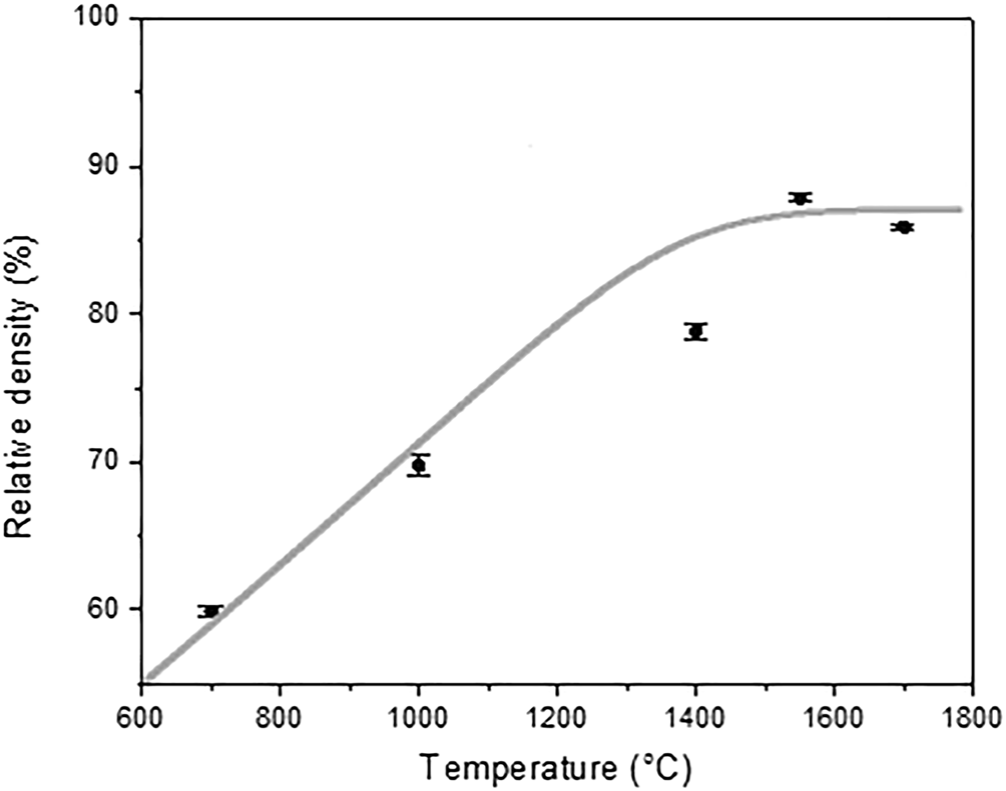
Evolution of the relative density of the SP-based printed cords at different temperatures.

Finally, relative density was measured at different temperatures considering 4.55 g/cm^3^ as theoretical density of YAG, see Fig. 8. At 700 °C, the relative density was about 60% due to the internal porosity and the uncomplete conversion of the xerogel into YAG. With the rise of temperature, organic phases are fully eliminated: for example, at 1000 °C, the relative density increased by 10%. This increment coincides with the full crystallization of the xerogel, nevertheless internal porosity remains. From 1400 to 1700 °C a noticeable improvement in the densification is appreciable. At 1400 °C, the relative density is around 80%. Then, at 1550 °C, cords reached the highest observed relative density of around 90% indicating that the packing of the grains was optimized, and the internal porosity was partially eliminated. Finally heating samples as high as 1700 °C, did not provide a further elimination of the internal porosity, but an activation of the grain growth.

## Conclusions

We successfully enlarged the production of YAG xerogel by modifying a protocol designed for a “laboratory scale” synthesis. Using dried YAG xerogel without prior pyrolysis as a solid load, a xerogel paste was formulated and then printed by robocasting. The printed structures of cords were calcined at different temperatures to monitor the transformation of the xerogel paste into a crystalline YAG ceramic. We have shown that it was possible to sinter and to obtain cohesive pieces after thermal treatments in the range of 1550–1700 °C, despite a partial remnant of the internal porosity.

Finally, the direct printing of xerogel paste without the usual prior pyrolysis, which implies larger organic departures, was not detrimental to the fabrication process. In addition, it reduces costs and would be appreciated by the industrial sector as an energy saving process. The fabrication of turbine parts for space exploration from YAG xerogel seems to be a promising approach.

## Methods

The structures of cords were extruded with a commercial 3D ceramic printer (Delta WASP 2040 clay) and a liquid deposit modeling extruder with a 1.2 mm diameter nozzle. Experimental conditions of 4 bars of compressed air flow, 4 mm/s printing speed, 1.5 mm high layers and a temperature of 20 °C with 50% of relative humidity (RH) were applied for all the extrusion tests. Green cords were dried at room temperature during 15 h in air with 50% RH.

The particle size distribution of the powders was measured with an LA-950 laser particle size analyzer (Horiba Ltd, Kyoto, Japan), in which a particle from the sample will scatter light at a defined angle determined by its size. A group of particles will thus produce a pattern of scattered light defined by its intensity and angle, which can be processed into a particle size distribution product. The measurements were carried out using the Fraunhofer-Kernel method, which is used to analyze the reflected and diffracted beams for the alumina particles.

XRD analyses for the powders and extruded cords were carried out with a Bruker-D8 Advance with a Bragg–Brentano geometry and a Cu K_α_ source, with an angular measurement range (2θ) of 15–90°, a step size of 0.012° and an equivalent time per step of 49.92 s. The identification of the crystalline phases refers to Joint Committee Powder Diffraction Standard (JCPDS) cards. The broadening of the highest peak (4 2 0) was measured to quantify the ordering degree of the crystalline YAG particles, with the help of a Voigt function taking into account the K_α1_-K_α2_ doublet of the source, which was used to determine the peak profile and extract its integral broadening.

Microstructures of the cords were observed with a scanning electron microscope (FEI quanta 450 FEG, Thermo Fisher Scientific, Eindhoven, The Netherlands) using a large field detector with a 5-kV beam voltage and a chamber pressure of 10^−5^ Pa. For the samples without thermal treatment, the extruded pieces were dried at room temperature for 72 h and then cut off to place them inside the sample holder. The samples were not metallized prior to observation. High-resolution captures were taken using a micro-imaging lens system Optem Fusion (camera mount 35-08-70-000) with a camera tube 35-41-10-000 and a fixed magnification of 12.5:1.

Thermogravimetric analyses (TGA) were conducted for the xerogels and paste with an SDT Q600, TA Instruments, where the samples were heated at 1200 °C in a platinum crucible at a heating rate of 5 °C/min under a dry airflow of 100 mL/min. It should be noted that every sample had an initial mass of approximately 50 mg.

The density of the powders was measured with a helium pycnometer (AccuPycII 1340, Micromeritics), in which the samples were placed into a 1 cm^3^ chamber. Helium gas was admitted and then expanded into another precision internal volume. The pressure before and after expansion was registered and it was used to measure the sample volume. This operation was repeated 10 times. On other hand, densities of the calcined 3D structures were evaluated employing the Archimedes’ principle, using deionized water and a digital analytical balance, operating with accuracy of 0.0001 gm. Density measurements were replicated three times and the average value was used to compare the different samples. Therefore, densities were calculating using the formula (1):

1 $$\rho = \frac{{m_{1} }}{{m_{1} - m_{2} }} \times \rho_{w}$$

where ρ is the density (g/cm^3^), m_1_ is the weight of the sample, m_2_ is the weight of the suspended sample inside of water-filled container and ρ_w_ is distilled water density (g/cm^3^).

## Supplementary Information

Supplementary Information.
