# Supplementary material for: Synthesis and robocasting of YAG xerogel: one-step conversion of ceramics
Source: Sci Rep. 2022 May 19;12:8454. doi: 10.1038/s41598-022-12204-6 (PMC9120145; doi:10.1038/s41598-022-12204-6)
Supplement: Supplementary file 1 — Supplementary Information. [file 41598_2022_12204_MOESM1_ESM.pdf]

SYNTHESIS AND ROBOCASTING OF YAG XEROGEL: ONE-STEP CONVERSION OF CERAMICS

Nancy Flores-Martinez[1,2], Lila Ouamara[2], Fabien Remondiere[2], Jenny Jouin[2], Giuseppe Fiore[1], Stephane Oriol[1], Sylvie Rossignol[2].

[1] Centre National d'Etudes Spatiales, Direction des Lanceurs, 52 rue Jacques Hillairet, 75615 Paris Cedex, France

[2] Institut de Recherche sur les Céramiques, UMR 7315, 12 rue Atlantis, 87068 Limoges Cedex, France

Corresponding author: sylvie.rossignol@unilim.fr

ADDITIONAL INFORMATION

Fig. AI-1 displays the thermal analyses showing the weight loss (%) and heat flow (W/g) of the xerogel (A) and the xerogel paste (B). In (A) an exothermal and endothermal event below 200 °C are related with the evaporation and elimination of volatile substances. Up to 720 °C, the oxidation of the organic molecules contained in the xerogel was finished. Around 780 and 910 °C, two exothermic events were observed, corresponding to the crystallization and arrangement of the xerogel into a garnet structure. A very similar behavior occurred in (B). Since, the formulation of the xerogel paste possessed a major amount of organic components; a well-resolved peak around 100 °C, corresponding an endothermic event, can be found around 100 °C. Then the elimination of the organic molecules took part in a broad exothermic lump up to 700 °C within which there are two small exothermic events at 370 and 540 °C. Finally, the last two exothermic shoulders around 780 and 910 °C matched with the reorganization of the structure previously mentioned in the xerogel powder. In addition, these two thermal events coincide with the crystallization and definition of peaks in the X-ray patterns of the cords.

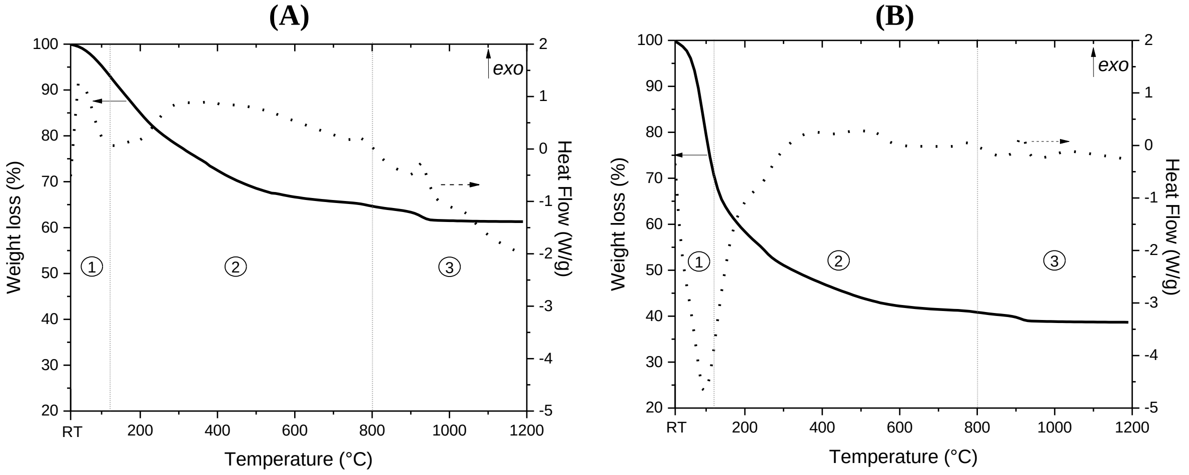

Fig. AI-1 Thermograms for (A) the YAG xerogel powder and (B) the YAG xerogel-based paste. Three zones can be distinguished corresponding to: ① evaporation, ② decomposition and ③ decarbonization processes.
